# Identification and Glycerol-Induced Correction of Misfolding Mutations in the X-Linked Mental Retardation Gene CASK

**DOI:** 10.1371/journal.pone.0088276

**Published:** 2014-02-05

**Authors:** Leslie E. W. LaConte, Vrushali Chavan, Konark Mukherjee

**Affiliations:** 1 Virginia Tech Carilion Research Institute, Virginia Tech, Roanoke, Virginia, United States of America; 2 Department of Biological Sciences, Virginia Tech, Blacksburg, Virginia, United States of America; CNRS UMR7275, France

## Abstract

The overwhelming amount of available genomic sequence variation information demands a streamlined approach to examine known pathogenic mutations of any given protein. Here we seek to outline a strategy to easily classify pathogenic missense mutations that cause protein misfolding and are thus good candidates for chaperone-based therapeutic strategies, using previously identified mutations in the gene CASK. We applied a battery of bioinformatics algorithms designed to predict potential impact on protein structure to five pathogenic missense mutations in the protein CASK that have been shown to underlie pathologies ranging from X-linked mental retardation to autism spectrum disorder. A successful classification of the mutations as damaging was not consistently achieved despite the known pathogenicity. In addition to the bioinformatics analyses, we performed molecular modeling and phylogenetic comparisons. Finally, we developed a simple high-throughput imaging assay to measure the misfolding propensity of the CASK mutants in situ. Our data suggests that a phylogenetic analysis may be a robust method for predicting structurally damaging mutations in CASK. Mutations in two evolutionarily invariant residues (Y728C and W919R) exhibited a strong propensity to misfold and form visible aggregates in the cytosolic milieu. The remaining mutations (R28L, Y268H, and P396S) showed no evidence of aggregation and maintained their interactions with known CASK binding partners liprin-α3 Mint-1, and Veli, indicating an intact structure. Intriguingly, the protein aggregation caused by the Y728C and W919R mutations was reversed by treating the cells with a chemical chaperone (glycerol), providing a possible therapeutic strategy for treating structural mutations in CASK in the future.

## Introduction

The X-linked mental retardation (OMIM300422 and OMIM300749) gene CASK codes for a MAGUK (Membrane-Associated Guanylate Kinase) scaffolding protein [1], [2]. The CASK protein is comprised of a CaM-kinase (Ca/calmodulin-dependent kinase) domain, two L27 (Lin2, Lin7) domains, a PDZ (PSD-95, Dlg, ZO-1) domain, an SH3 (Src homology 3) domain and a guanylate kinase (GuK) domain [1]. CASK domain arrangement is conserved in all metazoans, and individual domain structures exhibit a high degree of evolutionary conservation [3]. In vertebrates, the conservation of the primary structure is also very high [4]. Although CASK is ubiquitously expressed, its expression is strikingly high in the central nervous system, especially during development [5]. Mutations in CASK are associated with mental retardation as well as structural defects in the brain such as pontocerebellar hypoplasia, Ohtahara syndrome [6], FG syndrome with corpus callosum agenesis [6], [7], [8], [9], [10], tetralogy of Fallot [11], and autism spectrum disorders [12], [13].

Point mutations in the CASK gene are frequent in males with XLMR [8]. It is generally known that missense mutations can lead to pathogenesis via multiple mechanisms, including misfolding, mislocalization, defective expression, disruption of interactions, and change in function [14]. Chaperone-based therapeutics are a promising approach for pathologies associated with protein misfolding [15], therefore it is critical to develop simple and robust methods to classify structurally damaging mutations. The mechanisms by which point mutations in CASK cause a disease state are not clear. Here we have tested a combination of computational and in vitro approaches to more fully characterize five previously published pathogenic CASK mutations associated with neurodevelopmental disorder [7], [8] - R28L, Y268H, P396S, Y728C and W919R. There are a variety of computational programs available to predict whether a particular mutation is pathological, and we have used several to analyze the predicted impact of these mutations based on primary, secondary or tertiary structural information. To complement the computational analysis, we performed both a phylogenetic analysis and molecular modeling. To characterize these mutations in a cellular context, we expressed recombinant GFP-CASK mutants in cultured cells, allowing us to visually identify mutations that dramatically alter CASK’s subcellular distribution. The cell-based imaging assay provided a simple way to experimentally categorize mutations as either structural (causing misfolding) or functional (disruption of protein-protein interactions, alteration in enzymatic activity, etc.). The rationale for the observational evidence in these experiments is provided by our molecular modeling results. Our extensive assessment indicates that, due to a high degree of structural conservation among CASK orthologs, a phylogenetic analysis provides a simple and robust method to predict structurally damaging sequence variants and is more useful than any individual computational algorithm currently available. Finally, we demonstrate that a chemical chaperone may help rescue CASK that has misfolded due to a missense mutation. Overall our study provides a combination of a bioinformatics and experimental approaches to identify structurally damaging mutations in CASK which may be amenable to chemical chaperone-based therapeutic intervention.

## Materials and Methods

### Sequence-based Predictions of Mutation Effects

An analysis of phylogenetic conservation was performed based on a previously published alignment [4]. For all sequence-based bioinformatics analyses, CASK reference sequence NP_003679.2 was used. A conservation score for each mutation was calculated with the ConSurf web server [16] using the Uniref90 database, the BLOSUM62 matrix, and MUSCLE for multiple sequence alignment (MSA). An alternative MSA using 30 sequences identified during the ConSurf analysis (see Figure S1) was constructed using Clustal Omega v. 1.1.0 [17], submitted through the European Bioinformatics Institute (EMBL-EBI) webserver at http://www.ebi.ac.uk/Tools/msa/clustalo/ [18] using default settings. The MSA generated by Clustal Omega was then analyzed using Jalview [19].

The algorithms employed that predict protein stability changes based only on sequence were i-MutantDDG-Seq 3.0 [20] and ScPred [21]. Prediction of whether a particular mutation is pathogenic or neutral was performed using a variety of webserver-based algorithms: PhD-SNP v. 2.0.6 [22], PolyPhen-2, v. 2.0.22 [23], SIFT, v. 4.0.3 [24], SNAP, v. 1.0.8 [25], PON-P [26], and PMut [27].

The CASK sequence was assessed for regions of structural disorder via the PON-P webserver using DisProt VLS2 [28], GlobProt v. 2.3 [29], IUPred v. 1.0 [30], metaPrDOS v. 1.2 [31], and RONN 2007 [32]. Disorder predictions were done using CSpritz v. 1.1 [33], DISOPRED2 [34], FoldIndex [35], and PONDR-FIT [36]. PSIPRED was used to predict secondary structure for the region surrounding P396 [37], [38]. Aggregation propensities of residues in hCASK were assessed using the PASTA server [39]. Aggregation-prone regions were identified using the Aggrescan server [40], and the average aggregation propensity (Na4vSS; normalized sum of an amino acid aggregation propensity value over the entire input amino acid sequence) was calculated for each of the 5 mutations of hCASK.

### Molecular Modeling

Molecular visualization, editing and analysis was done and publication images were produced using the UCSF Chimera package [41].

Modeller9.9 was used [42], [43] to construct a molecular model of hCASK that included residues 728 and 919. Within Modeller, the salign command was used to generate an alignment between the C-terminal portion of the hCASK sequence starting at residue 614 (encompassing the SH3 and GuK domains) with the two template structures, 1kgd (hCASK GuK domain) and 1 kjw (PSD-95 SH3-GuK domain) that had been modified using the Modeller DOPE_loopmodel command to account for residues that were not included in the crystal structures. The automodel command was used to generate 10 homology models; the molecular model with the lowest DOPE score that did not have knots was used for further analysis.

Structural models of hCASK with mutations of interest were constructed using Chimera’s Rotamers structure editing tool (residues 28 and 268 in 3c0i.pdb and the equivalent of residues 728 and 919 in the hCASK SH3-GuK molecular model). Rotamers from the Dunbrack backbone-dependent rotamer library [44] with the highest probability and least number of clashes were selected.

Contacts and clashes were calculated in Chimera using the Structural Analysis tool, “Find Clashes/Contacts”. Contacts were defined as pairs of atoms with a separation between their van der Waals radii of less than or equal to 0.4 Å, ignoring intra-residue contacts and contacts of pairs that are 4 or fewer bonds apart. Clashes were defined as pairs of atoms with an overlap of their van der Waals radii of at least 0.6 Å, with a hydrogen bond allowance factor adjustment of 0.4 Å.

### Structure-based Predictions of Mutation Effects

The published structure of CASK’s CAMK domain (3c0i.pdb) and our molecular model of CASK’s SH3-GUK domain were used for all algorithms requiring structure data. Web server-based PoPMuSiC [45], [46], Eris [47], and CC/PBSA ([48] were used to assess changes in free energy (ΔΔG) between wild-type and mutant CASK sequences. PoPMuSiC was used to calculate solvent accessibility. The command-line program FoldX 3.0 Beta 5.1 [49] was used to assess protein stability. Wild-type structures were minimized using the ‘RepairPDB’ command, and FoldX’s built-in mutagenesis engine was implemented with the ‘BuildModel’ command to make mutations and calculate ΔΔG values.

### Plasmid and Point Mutagenesis

**Figure 1:**
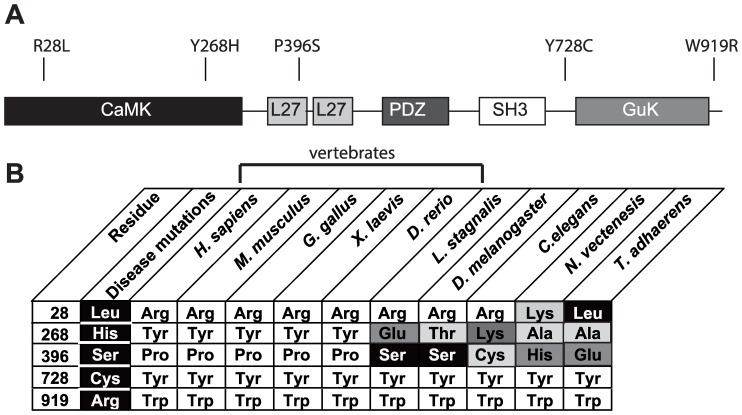
Domain location and conservation of five CASK mutations. *A.* Five CASK XLMR mutations are shown in reference to CASK’s domain structure. *B.* A comparison of the five mutation sites in CASK orthologs from nine species. Conserved residues, white. Residues identical to the mutation, black. Residues that differ from the wild-type and mutant hCASK sequence are gradiently shaded to indicate their similarity to the native hCASK residue.

CASK was cloned in the pEGFP-C3 vector as previously described [50]. Site-directed mutagenesis was performed using Phusion polymerase (NEB), creating point mutations R28L, Y268H, P396S, Y728C and W919R in the human CASK gene (see Figure 1A and Figure S2) and sequenced in the Core Laboratory Facility in the Virginia Bioinformatics Institute at Virginia Tech.

### Cell Culture and Imaging

Human embryonic kidney (HEK-293) cells (ATCC) were plated on 50 µg/ml poly-L-lysine (Sigma Aldrich Inc.)-coated coverslips (Fisherbrand, Inc.) in 24-well plates (JetBiofil) and maintained in DMEM (Hyclone) containing 10% fetal bovine serum (Hyclone) supplemented with 5 mg/ml penicillin-streptomycin (Hyclone). Cells at 80% confluency were transfected with 0.25 µg of GFP-CASK DNA per well using calcium phosphate. Six hours post-transfection, fresh media was exchanged containing either no glycerol or 10% glycerol. Twenty hours post-transfection, cells were washed twice with phosphate buffered saline (Sigma Inc.) and fixed for 15 minutes at room temperature using a 4% paraformaldehyde solution. Coverslips were mounted on microscope slides (Premiere) using Vectashield (Vector Laboratories Inc.) and visualized using confocal laser scanning microscopy (ZEISS Axio Examiner.Z1 LSM 710). The proportion of transfected cells with visible aggregates from three independent experiments were counted using the cell counter plugin of the Image J program (http://imagej.nih.gov/ij/; [51]). For each condition, five high-power field images were analyzed for aggregation. Total cells and cells containing aggregates were visually identified and tallied. Percent of total cells containing aggregates was then calculated. The image analysis procedure was repeated 3 times for each condition and averaged.

Co-transfection with CASK and mCherry was done as described above, with 0.5 µg of mCherry plasmid DNA added to each well of a 24-well plate in addition to the desired CASK DNA (Y728C or W919R). For thioflavin T staining, cells on coverslips were washed and fixed as above and were then incubated in 200 µL of a 0.05% thioflavin T (Acros Organics) solution in 0.1N HCl for 8 min in the dark. Coverslips were then mounted as above. To co-express CASK and CellLight® Golgi-RFP, BacMam 2.0 (Life Technologies), the CASK (Y728C or W919R) transfection was done as described above. After 6 hours, cells were washed, and 15 µL of CellLight® Golgi-RFP in media was added to each well of a 24-well plate. Imaging was performed after 48 hours of incubation.

### Co-immunoprecipitation

HEK-293 cells were plated in 6-well plates (JetBiofil) and maintained as described above. Cells at 80% confluency were transfected with 2 µg of wild-type or mutant (R28L, Y268H, P396S) GFP-CASK DNA per well and co-transfected with 2 µg liprin-α3 and/or Mint-1 (FLAG-tagged) DNA in each well using calcium phosphate; native Veli from HEK cells was used for the CASK-Veli co-immunoprecipitation experiment. Six hours post-transfection, fresh media was exchanged. After 48 hours, cells were collected by adding 500 µl chilled PBS and centrifuged at 2350 g, 4°C for 10 min. Cell pellets were frozen at −20°C until being processed for immunoprecipitation.

Cell pellets were resuspended in 500 µL lysis buffer containing PBS, 2 mM DTT, 2 mM EDTA and protease inhibitors. Pellets were uniformly homogenized by using a VWR™ Pellet Mixer. One percent Triton-X was added to the homogenized sample, which was centrifuged for 15 min at 21,100 g and 4°C. Supernatants were collected. Five microliters of Chromotek GFP-Trap® agarose beads per sample were washed twice with 100 µL lysis buffer. Beads were then incubated with the supernatants at 4°C on a rocker for 1 hour and centrifuged for 2 min at 2350 g, 4°C. Pellets were washed twice with lysis buffer, and 5 µL of 2x sample buffer was added to each sample. Samples were boiled at 100°C for 10 min before loading them on 10% polyacrylamide gels for subsequent western blotting (wet transfer) for 2 hours at 80V onto a nitrocellulose membrane (Whatman Protran BA 85). Blots were incubated with either anti- liprin-α3, anti-velis (gifts, Thomas Südhof), or anti-FLAG (Sigma Inc.) primary antibodies in blocking buffer (5% powdered milk in wash buffer, below) for 1 hour at room temperature with rocking. After washing three times (in wash buffer; 150 mM NaCl, 0.05% Tween, and 20 mM Tris, pH 7.2), blots were incubated with goat anti-rabbit (liprin-α, Velis) or goat anti-mouse (anti-FLAG) HRP conjugate (ImmunoReagents Inc.). Blots were developed using Amersham ECL western blotting detection reagents (GE Healthcare Life Sciences) and imaged using a ChemiDoc™ MP System (BioRad).

## Results

In this study, five missense CASK mutations associated with variable XLMR phenotypes [7], [8] were subjected to both in silico and experimental analysis to better understand the underlying disease-causing mechanism. Results for each mutation are presented below, detailing information about each residue’s phylogenetic conservation, outcomes from sequence-based bioinformatics tools, information derived from available structural models, as well as images of GFP-CASK and its mutants in cell culture and in preliminary immunoprecipitation studies. In the current manuscript we analyzed five known missense mutations in CASK associated with neurodevelopmental disorders: R28L, Y268H, P396S, Y728C and W919R.

### Domain Location and Phylogenetic Analysis of CASK Missense Mutations

R28 resides in the CaMK domain of hCASK. An arginine at this particular location is highly conserved throughout many metazoans (Figure 1). Changes at position 28 from arginine are present in CASK orthologs; in fact, in a representative Placozoan species (T. adhaerens), residue 28 is a leucine, the residue in hCASK identified as a mutation that causes FG syndrome [7]. Y268 also resides in the CAMK domain of hCASK. CASK sequence conservation (Figure 1B) suggests that a tyrosine is typically found at this position in vertebrates but is not conserved outside of this phylum. At position 396, located in the first L27 domain of hCASK, a proline is commonly observed in vertebrates (Figure 1B), but outside the Chordate phylum, a range of residues is observed, including serine, the mutation associated with XLMR [8]. The residues Y728 and W919 flank the guanylate kinase (GuK) domain in the C-terminus of CASK. These residues are highly conserved across phyla (Figure 1B).

### Sequence-based Prediction of Pathogenicity of Mutations

In addition to a simple visual examination of mutation sites using a CASK alignment, as described above, more robust bioinformatics analyses can be performed on sequence alignments of CASK orthologs. ConSurf [16] and Jalview [19] are both examples of programs that calculate a conservation score based on a multiple sequence alignment (MSA) of orthologous proteins. The ConSurf score represents the rate of evolution at a specific site and can range from 1 (highly variable) to 9 (highly conserved). Additionally, ConSurf calculates the frequency of each type of amino acid observed at a specific site. ConSurf returned a conservation score of 6 for position 28 (Table 1), indicating some degree of conservation. Additionally, at position 28, an arginine was observed in 81% of the sequences, a lysine was observed in 14% of the sequences, and a leucine (the hCASK mutation of interest) was observed 5% of the time. Jalview analysis of a Clustal Omega MSA yielded a conservation score of 0 for position 28. For the Y268 position, both the ConSurf score (1) and Jalview conservation score (0) (Table 1) confirm that this is a highly variable site. P396 is also predicted to be highly variably by both ConSurf and Jalview (Table 1), and interestingly, the ConSurf analysis reveals that of the 30 sequences included in the alignment, 23% have a serine at that site and only 13% have the proline found in hCASK. The only sites predicted by ConSurf and Jalview to be highly conserved are Y728 and W919 (Table 1), the same sites of interest from the initial visual examination (Figure 1B). Based on the sequence, ConSurf classifies both Y728 and W919 as buried and structural and therefore likely to be important for structural stability.

**Table 1:** Predicted impact of mutations (sequence-based).

	Sequence Conservation		Stability Change		Overall Pathogenesis					
Mutant	ConSurf Score	Jalview Score	I-Mutant 3.0	SCPred	PhD-SNP	Poly Phen-2	SIFT	SNAP	PON-P	Pmut
R28L	6	0	N	yes	N	P	N	P	P*	N
Y268H	1	0	P	no	N	N	N	P	N	N
P396S	1	0	P	yes	N	N	N	N	N	N
Y728C	9	11	N	no	P	P	P	P	P	P
W919R	9	3	P	yes	P	P	P	P	?	P

ConSurf conservation scores: 1 (variable) to 9 (highly conserved). Jalview conservation scores: 0 (variable) to 11 (highly conserved). SCPred: residue serves as a stabilization center (“yes”) or not (“no”). N = neutral, P = pathogenic, ? = unclassified. *prediction unreliable at 0.95 probability level.

Many algorithms incorporate sequence conservation in their methodology for predicting whether a particular mutation will be pathogenic or not. Three algorithms (PolyPhen-2, SNAP, and PON-P; Table 1) categorized R28L as pathogenic. Three different algorithms (PhD-SNP, SIFT, and Pmut), however, classified the R28L variant as neutral or not pathogenic. Algorithms that predict only whether or not a mutation affects the stability of a protein’s fold were also ambiguous; I-Mutant predicted that R28L would have no impact, whereas the SCPred algorithm classified R28 as a stabilization center, suggesting that interactions made by this residue must be broken in order for the protein to unfold. For the Y268H mutation, most algorithms predicted the impact of such a sequence change would be neutral, with the exception of SNAP (Table 1) and I-Mutant, which predicted that this mutation would destabilize the protein. Sequence conservation-based algorithms predicted that a P396S mutation will be neutral in terms of pathogenicity (Table 1), but I-Mutant and SCPred predicted that this mutation will negatively impact protein stability. Y728 and W919, easily identified as highly conserved as described above, were consistently predicted to cause pathogenesis, although Y728C was not predicted to cause a change in protein stability (Table 1).

### Structure-based Prediction of Pathogenicity of Mutations

**Figure 2:**
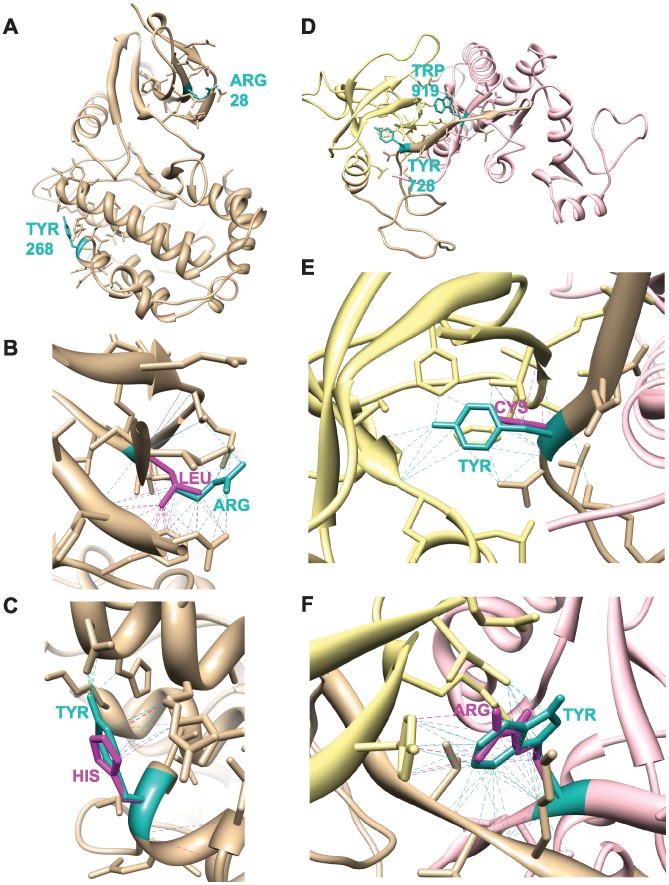
Structural modeling of four CASK mutations. Dotted lines indicate contacts. *A.* CAMK domain of CASK (3c0i.pdb) showing R28 and Y268. *B.* Native (Arg, cyan) and mutant (Leu, magenta) side-chains at position 28. *C.* Native (Tyr, cyan) and mutant (His, magenta) side-chains at position 268. *D.* SH3-GuK domain homology model showing Y728 and W919. SH3 region, yellow. GuK region, pink. *E.* Native (Tyr, cyan) and mutant (Cys, magenta) side-chains at position 728. *F.* Native (Trp, cyan) and mutant (Arg, magenta) side-chains at position 919.

The published structure of CASK’s CAMK domain (3c0i.pdb) and a molecular model of CASK’s SH3-GUK domain were used to predict the impact of each mutation on overall protein stability using algorithms that require structural input. R28 is on the surface of the protein (Figure 2A) and considered an exposed residue by ConSurf. The R28L mutation slightly reduces the number of contacts (Figure 2B, Table 2). Calculations of ΔΔG (kcal/mol) with the R28L mutation were performed using this structure. An increase in ΔGmut (positive ΔΔG) suggests that a mutation destabilizes the protein fold. For R28L, both Eris and CC/PBSA predict destabilization (Table 2). PoPMuSIC and FoldX predict that the mutation is, however, stabilizing. Bioinformatics efforts to predict the impact of the R28L mutation on structure were thus inconclusive. Y268 is on the surface of the protein (Figure 2A), is considered an exposed residue by ConSurf, and is even more solvent-accessible than R28L (Table 2) according to PoPMuSiC. The Y268H mutation decreases the number of contacts (Figure 2C; Table 2). All methods that predict ΔG suggest that the Y268H mutation slightly destabilizes the overall protein fold (Table 2).

**Table 2:** Predicted impact of mutations (structure-based).

	Chimera	PoPMuSIC		FoldX	Eris	CC/PBSA
Mutant	Change in numberof contacts	Solvent accessibility(WT residue)	ΔΔG(kcal/mol)*	ΔΔG(kcal/mol)*	ΔΔG(kcal/mol)*	ΔΔG(kcal/mol)*
R28L	−5	16.80%	−0.28	−1.65	1.3	1.01
Y268H	−10	35.05%	0.5	1.83	1.17	2.11
Y728C	−12	4.22%	2.77	4.57	6.9	4.32
W919R	−14	18.40%	2.1	3.51	−0.38	4.05

For mutations in CaMK domain (R28L and Y268H), the structure 3c0i.pdb was used. For mutations in the SH3-GuK domain (Y728C and W919R), the homology model based on 1 kgd.pdb and 1 kjw.pdb was used. Positive ΔΔG values suggest that the indicated mutation destabilizes CASK’s overall fold.

P396 is located at the end of the first L27 domain of hCASK near the junction between CASK’s two L27 domains. NMR structures of isolated L27 domains from SAP97, CASK, and LIN-7 in various dimers have been published [52], [53], [54], but none of these complexes contain P396S because the linker region between CASK’s two L27 domains is not included in any of the NMR structures. The lack of a structure containing P396 precluded use of algorithms based on structural analysis. However, the majority of algorithms that use sequence to predict whether a residue is in an ordered or disordered portion of a protein (DisProt, FoldIndex, GlobProt, IUPred, PONDR-FIT, RONN) predict that P396 is in a disordered segment that ranges from 10 to 18 residues in length. The other mutations (R28L, Y268H, Y728C, and W919R) were confirmed to be in ordered regions, as expected given the known structures of CASK domains where they are located. A few algorithms (DISOPRED2, MetaPrDOS, and CSpritz), however, predict that P396 is in an ordered region of hCASK, although the DISOPRED2 number is much closer to the disorder threshold than any of the other residues. The secondary structure prediction algorithms PSIPRED [37] and CSpritz predict neither helix nor beta sheet properties for this region of CASK.

The crystal structure of the GuK domain of hCASK (1 kgd) [55] does not include Y728 or W919. We therefore constructed a molecular model of hCASK that spans the SH3 and GuK domain (Figure 2D), based on 1 kgd and the published crystal structure of PSD-95 (1 kjw; [56]), which, like CASK, is a MAGUK scaffolding protein and structurally homologous to the C-terminus of CASK (25.9% identity and 48.4% similarity, as calculated by EMBOSS_Matcher [57] run on the EMBL-EBI server; http://www.ebi.ac.uk/Tools/psa/emboss_matcher/). W919 and Y728 are located in adjacent beta strands that comprise an integrated SH3-GuK domain characteristic of MAGUK proteins [56]. Y728 is predicted by PoPMuSiC to have very low solvent accessibility, and W919 is predicted to be more solvent-accessible (Table 2). Based on this SH3-GuK model, both the Y728C and W919R mutations decreased the number of contacts (Table 2). PoPMuSiC and FoldX both predicted positive ΔΔG values for Y728C and W919R (Table 2). Eris predicted a large decrease in protein fold stability for Y728C, but a slight increase in protein fold stability for the W919R mutation (Table 2).

### Cytosolic Behavior of CASK Mutants and Protein-protein Interaction

**Figure 3:**
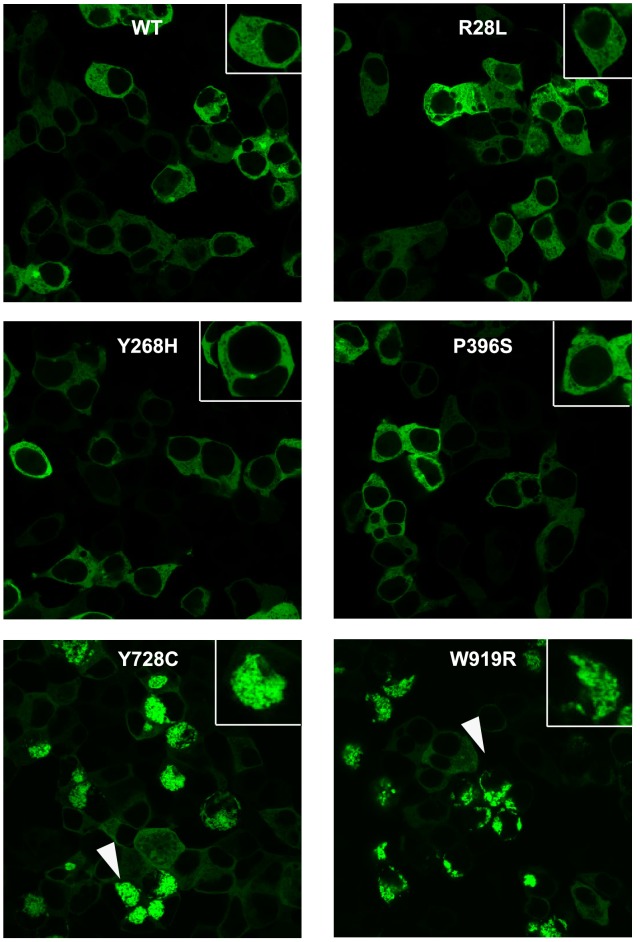
Subcellular localization of GFP-hCASK and GFP-hCASK mutants in HEK-393 cells. Images were obtained with a 63X Plan-apochromat 1.4 N.A oil lens. White arrows indicate representative intracellular aggregates. Insert shows higher magnification.

The various bioinformatics analyses performed did not provide unequivocal predictions for the CASK mutations. The most consistent pathogenicity predictions were achieved for the mutations in the invariant residues Y728 and W919. To further investigate these predictions, we developed a simple cell biological assay. Wild-type GFP-CASK, when expressed in HEK cells, displays a diffuse cytosolic localization pattern (Figure 3 and Figure S3). When R28L-GFP-hCASK was introduced into HEK cells (Figure 3), the intracellular distribution was indistinguishable from wild-type, appearing to be uniformly distributed throughout the cytoplasm and excluded from the nucleus. This observation suggests no large aggregates are formed by R28L-CASK. Similarly, when Y268H- and P396S-GFP-hCASK were studied in the cellular context, there was no apparent difference in distribution or solubility from GFP-CASK (Figure 3). Y728C- and W919R-GFP-hCASK, however, do not exhibit the same cellular distribution pattern in HEK cells that is observed with wild-type GFP-hCASK (Figure 3). Both Y728C-GFP-CASK and W919R-GFP-CASK, instead of being uniformly diffuse throughout the cytoplasm, are typically concentrated in a perinuclear region on one side of the cell, suggesting potential protein aggregation. There was no significant difference in overall protein solubility between wildtype CASK and either of these mutants when blotting for CASK in the soluble and insoluble fractions of cell lysate (Figure S4).

Computational methods are capable of predicting regions of a protein that may be involved in forming amyloid fibrillar aggregates. Because of the observed changes in CASK solubility with Y728C and W919R (Figure 3), we examined the CASK sequence for aggregation-prone regions that could lead to fibril formation if exposed due to structural destabilization. The Aggrescan server [40] predicts “hot spots” of aggregation potential within a protein sequence, as well as the impact of a mutation on a protein’s aggregation propensity. The PASTA server [39] identifies stretches in protein sequences prone to β-aggregation. CASK’s PASTA aggregation profile identified two regions with notable aggregation propensity: residues 207–213 in a helix in the CaMK domain of hCASK, and residues 853–859 in a beta strand in the GuK domain of hCASK. Aggrescan also identified these two regions as aggregation “hot spots.” None of the XLMR mutations studied, including Y728C and W919R, changed hCASK’s aggregation profile as calculated by PASTA or Aggrescan (data not shown).

**Figure 4:**
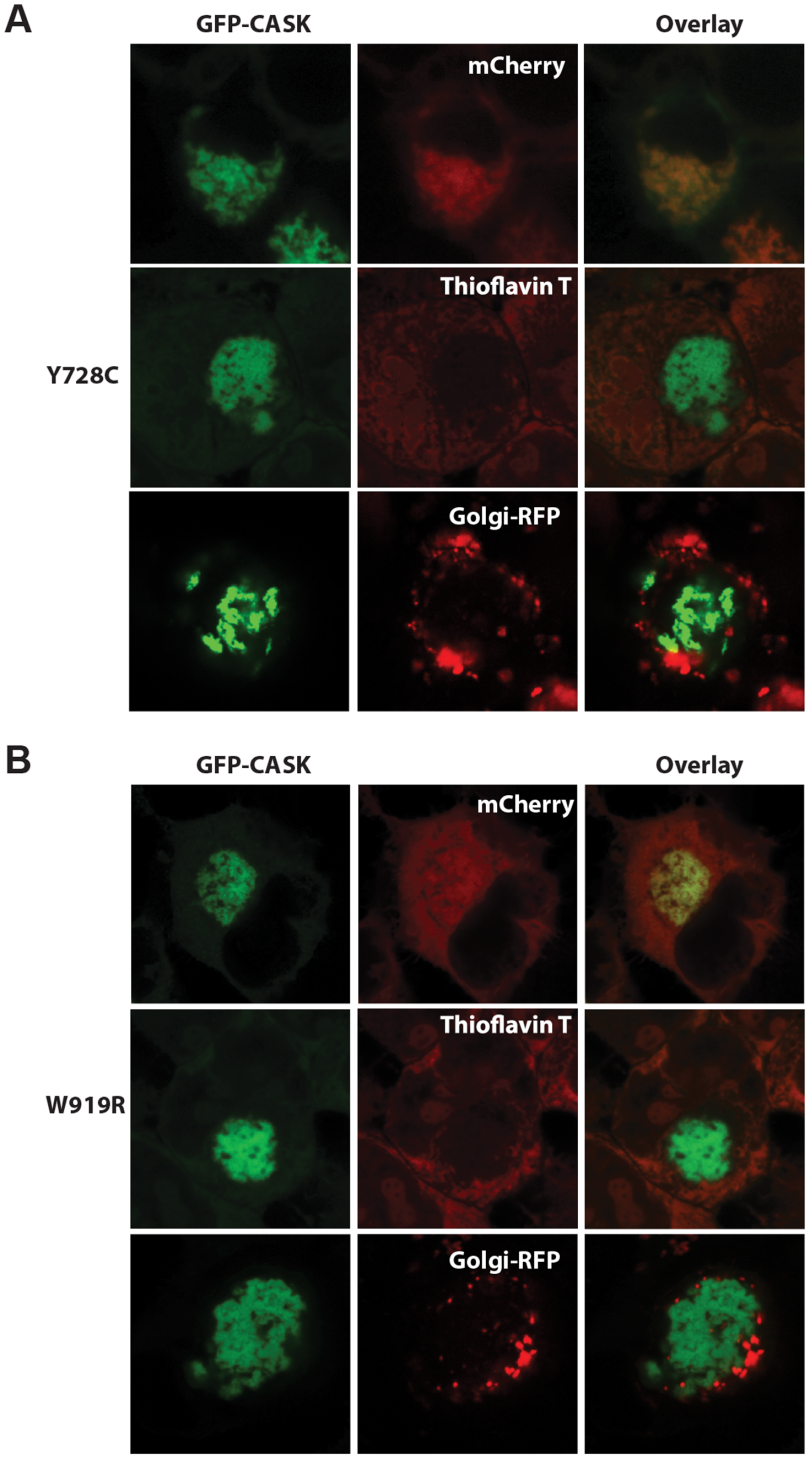
Characterization of aggregates. Images of HEK cells transfected with *A)* GFP-CASK-Y728C or *B)* GFP-CASK-W919R were obtained with a 63X Plan-apochromat 1.4 N.A oil lens. First column shows aggregated GFP-CASK protein. Panels labeled “mCherry” show cells that were co-transfected with GFP-CASK and mCherry, which remains cytosolic. Panels labeled “Thioflavin T” represent coverslips that were fixed and then stained with Thioflavin T, which shows enhanced fluorescence in the presence of amyloid fibrils. Panels labeled “Golgi-RFP” represent coverslips that were treated with CellLight® Golgi-RFP which labels the Golgi network. Third column shows an overlay, demonstrating that aggregates are cytosolic (mCherry, Golgi-RFP) but not amyloid in nature (Thioflavin T).

To further character the aggregates seen in cells expressing CASK containing either the Y728C or W919R mutation, additional imaging experiments were done. To rule out the possibility that the other mutations (R28L, Y268H, and P396S) also aggregated, but on a longer timescale, cells transfected with all forms of mutant CASK were imaged after waiting 72 hours (rather than 20 hours) after transfection (Figure S5). First, cells were co-transfected with the mutated CASK of interest (either Y728C or W919R) and with mCherry, a fluorescent protein known to be cytosolic in nature [58]. In cells co-transfected with CASK containing either mutation, mCherry co-localizes with the aggregated CASK, confirming that CASK is not trapped in a membrane-bound cellular compartment such as the endoplasmic reticulum or Golgi but is indeed cytosolic (Figure 4). To further demonstrate the cytosolic nature, we co-transfected the mutant GFP-CASKs (either Y728C or W919R) with CellLight® Golgi-RFP, which specifically labels the Golgi network [59]. These images suggest that GFP-CASK aggregates do not localize to these membrane-bound organelles. To determine whether the aggregates were amyloid in nature, Thioflavin T staining was performed on cells expressing CASK with either of the mutations of interest. Upon Thioflavin T binding to amyloid fibrils, the fluorescence of Thioflavin T is enhanced and undergoes a red shift and can thus be used to identify amyloid fibrils in cell culture [60]. As is evident in Figure 4, the aggregates formed by CASK mutants in cell culture are not stained by Thioflavin T, providing evidence that these aggregates are not composed of amyloid fibrils. When a ubiquitin antibody was used to immunostain cells, the aggregates did not show any specific staining (data not shown), suggesting that the aggregates do not contain markedly high levels of ubiquitin.

**Figure 5:**
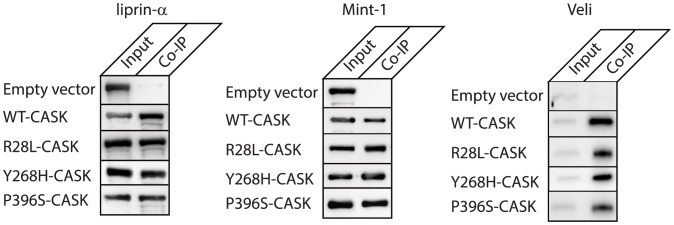
Functional CASK XLMR mutations (R28L, Y268H and P396S) do not disrupt interactions with liprin-α, Mint-1, or Veli. Lysates from HEK-293 cells co-transfected with GFP-CASK (wild-type or mutants R28L, Y268H, or P396S) and either liprin-α3 or FLAG-tagged Mint-1 were incubated with anti-GFP beads to pull down GFP-CASK and binding partners. To assess Veli interaction, no co-transfection was performed; native Veli was pulled down after incubation of lysates from GFP-CASK-transfected HEK-293 cells with anti-GFP beads to pull down GFP-CASK. Western blots of samples containing whole cell lysate (Input) or immunoprecipitates (Co-IP) were probed with anti-liprin-α3, anti-Veli, or anti-FLAG primary antibodies.

Based on the results presented above, it seems likely that the R28L, Y268H and P396S mutations are structurally stable and may instead result in pathogenesis because of functional disruptions such as aberrant or absent protein-protein interactions. Protein interactions with binding partners can be used as functional probes for detecting alterations in 3D structure. The CaMK domain of CASK, where R28L and Y268H are located, is involved in interactions with Mint-1 and liprin-α [61], [62], and the L27 domain of CASK, where P396S is located, is the site of Veli binding [61]. Immunoprecipitation experiments suggest that the previously characterized interactions of the CaMK domain with liprin-α [62] and Mint-1 [61] are not disrupted by either the R28L or Y268H sequence variants (Figure 5). Similarly, the interaction between CASK and Veli [61] is preserved in the presence of the P396S mutation (Figure 5). The maintenance of the R28L-CASK interaction with liprin-α is consistent with an earlier study [7]. It had been concluded earlier that the Y268H mutation, however, weakened the CASK-liprin-α interaction [62]; this was not observed in our co-immunoprecipitations (Figure 5). Because it has been shown that phosphorylation of the serine immediately following P396 is critical for synaptic recruitment of CASK and its interactions with liprin-α [63], we decided to test the interaction of P396S-CASK with liprin-α and Mint-1. Our data indicates that both interactions are intact when CASK contains the P396S mutation, as is the interaction between CASK and Veli, despite the location of this mutation in the L27 domain known to be the site of this interaction.

### Glycerol as a Chaperone for Protein Folding

**Figure 6:**
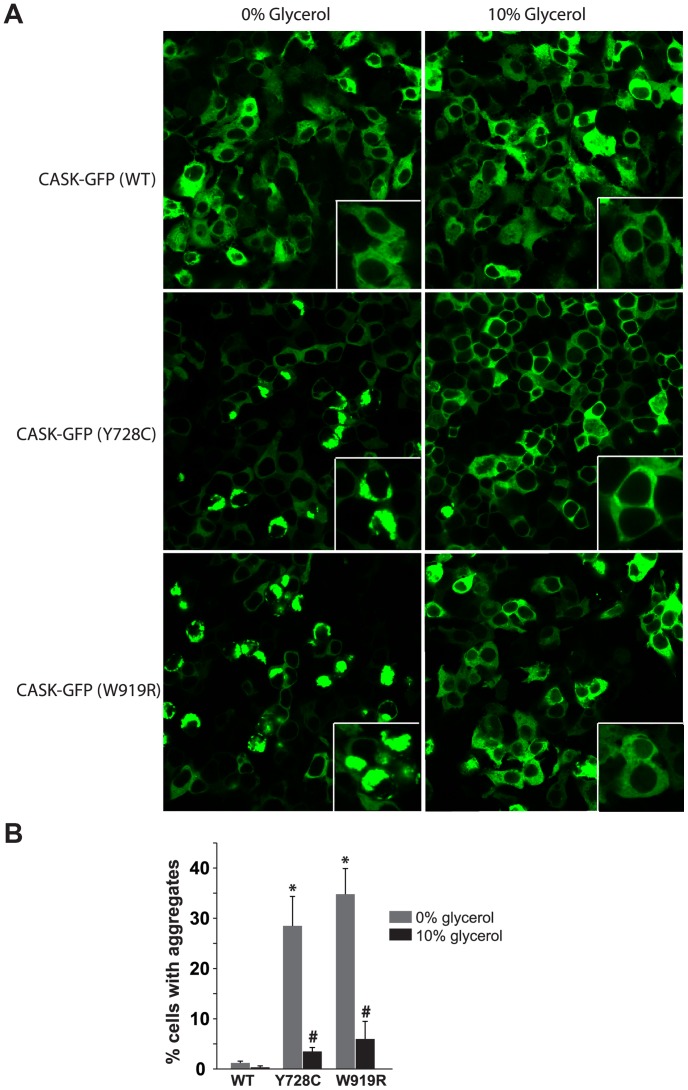
Glycerol treatment eliminates intracellular aggregates. Six hours after transfection, media was exchanged for either fresh media alone or containing 10% glycerol. *A.* Images, 40X. Insert shows higher magnification. *B.* Using five representative 20X images (Figure S7) for each condition, individual cells were classified as free of or containing aggregates in Image J. Bars and error bars represent the average and standard deviation of three independent analyses. * and # indicate statistically significant differences from the wild-type images.

As a chemical chaperone, glycerol can both stabilize beta fibrils, enhancing beta aggregation [64], and correct denaturation in mutant forms of proteins like CFTR [65] and aquaporins [66] (reviewed in [67]). Since our analysis suggests that the aggregation caused by Y728C and W919R is not fibrillar in nature but rather represents misfolded proteins, normal media was supplemented with 10% glycerol following transfection to inhibit the apparent aggregation observed with Y728C and W919R hCASK. Incubation in glycerol-supplemented media did decrease cell viability somewhat (approximately 20%; Figure S6). Importantly, however, glycerol supplementation significantly reduced visible GFP-CASK aggregation (Figure 6A), and in the majority of glycerol-treated cells (Figure 6B), Y728C and W919R GFP-CASK exhibited a distribution similar to WT GFP-CASK (images showing a broader sampling of cells are included in Figure S7).

## Discussion

The accelerated discovery of disease-associated missense sequence variants necessitates the development of high-throughput pipelines, both experimental and bioinformatic, to understand the underlying mechanism of pathogenicity. Many algorithms have been developed to assess the potential for a protein point mutation to cause pathogenesis broadly defined, however, without simple experimental screens, these predictions cannot be easily verified. Of greater importance for designing rational, mutation-specific therapeutic strategies for a particular disease is determining whether a given missense mutation causes a change in protein function or results in protein misfolding and aggregation. Pathologies associated with protein misfolding can be potentially treated with molecular or chemical chaperones [15].

Missense mutations in CASK are frequently identified in boys with mental retardation [8] and are often associated with structural defects in the brain, as well as the head, neck, and face region. We chose to examine five CASK missense mutations that, based on genetic analysis, are associated with disease state but whose impact on CASK structure and/or function has not been definitively determined. We predicted that these mutations would cause pathogenesis based on a loss of CASK function either due to global misfolding or changes in protein-protein interaction. To both elucidate the pathogenic mechanism generated by a particular mutation, as well as to consider more broadly the techniques available to assess the impact of missense sequence variants on proteins, we examined these mutations in silico and in cell culture.

Of the five mutations analyzed here, only two are categorically invariant, indicating that mutations in the other three residues might be structurally tolerated. Two of these (R28L and P396S) correspond to native residues in known CASK orthologs (Figure 1B). Our data supports evidence in the literature that the R28L and Y268H mutations do not affect the overall structure of CASK [7], [62]. Computational algorithms employed, whether sequence-based or structure-based, offered no consensus about the neutrality of the R28L mutation. The cell-based study (Figure 3), however, suggests that there is no change in subcellular localization or folding, and a dramatic decrease in R28L-hCASK protein stability seems highly unlikely because this variant already exists in nature (Figure 1). The mechanism behind the XLMR disease state associated with the R28L mutation is thus unlikely to be due to protein misfolding. A previous study of R28L-hCASK indicated that although a majority of its transcripts were normal, the mutation resulted in a small fraction of transcripts that were missing exon 2 [7]. Whether a CASK product lacking exon 2 is toxic, or is even produced, is not clear. Our co-immunoprecipitation results suggest that the R28L mutation does not disrupt CASK’s interaction with either liprin-α, Mint-1, or Veli (Figure 5), although other functional impacts of this sequence change remain to be explored.

There is strong evidence to suggest that CASK evolved as a result of the incorporation of a CaMK domain into an ancestral MAGUK protein [4]. A comparison of CASK’s CaMK domain sequence with representative sequences of CaM kinase I and CaM kinase II [68] offers further insight into the R28L hCASK mutation. Like hCASK, an arginine is typically found at this position in CaM kinase II, whereas in CaM kinase I, there is a leucine that plays a role in autoinhibition by interacting with the regulatory domain [68]. This strengthens the conclusion that a leucine is tolerated within this particular protein fold and has broader implications regarding the possible importance of this residue in the autoinhibition of the kinase activity of this domain, which may explain the alteration in protein function that results in XLMR associated with R28L-CASK.

Y268 is conserved only in vertebrate CASK (Figure 1). This tyrosine is located in the last alpha helix of a catalytic core that is conserved across a variety of protein kinases [68]. As an exposed residue (Figure 2C) with low sequence conservation, it is unlikely to cause folding problems [69], as supported by both our computational and cell-based findings (Table 2; Figure 3). The pathogenicity associated with the Y268H mutation is thus more likely functional. Y268H has been shown to reduce affinity towards CASK’s interacting partner, liprin-α [62], although this was not convincingly demonstrated by the co-immunoprecipitation experiments performed here (Figure 5). The liprin-CASK interaction seems to be vertebrate-specific [70], [71], which fits well with the observation that Y268 conservation is only seen in vertebrates. Although much evidence suggests that liprin-α is a key player in presynaptic active zone assembly [72], [73], it is important to note that most synaptic data comes from invertebrate animal models [70], [74], [75]. Whether the vertebrate-specific CASK-liprin interaction has additional synaptic roles is not clear, although a recent study in mammalian neurons suggests that depletion of liprin-α2 at mature synapses results in less synaptic localization of CASK [72]. Deletion of CASK in mice has little effect on synapse formation or structure [76]. Interestingly, liprin’s interaction via CASK’s CAMK domain suggests that it may compete with other proteins that interact at this site, such as Caskin [77] and the evolutionarily conserved CASK-Mint1 interaction [61]; the mutation at position 268 might thus disrupt CASK’s affinity for liprin-α enough to shift the predominant binding partner in vivo.

Much evidence suggests that the P396S mutation does not affect hCASK’s ability to fold. The fact that serine is frequently found at this position in phyla outside of Chordata (Figure 1B) suggests not only that a serine is tolerated at this position but that the shift from serine to proline might play a defining role in the function of this protein in chordates. The location of this residue in a linker region between CASK’s two L27 domains leads to the speculation that this mutation may affect the ability of the protein to undergo domain rearrangements that are necessary for function. L27 domain-dependent hetero-oligomerization between scaffolding proteins may be critical in synaptic signaling [52]. The fact that XLMR is associated with the elimination of a proline at this position in hCASK led us to more closely consider the critical role proline can play in protein structure. Proline cis-to-trans isomerism has previously been proposed as a gate-keeper for fibril formation [78], [79] and can also serve as a molecular switch, as is seen in the pores of some neurotransmitter-gated channels [80], [81]. It is possible that the isomerization state of P396 is an important regulator of protein-protein interactions that involve hCASK’s L27 domain and hence hCASK cellular function, and when a proline is lost at position 396, a yet-to-be-described function of hCASK is lost.

The two XLMR mutations that flank CASK’s GuK domain (Y728C and W919R) were the only two CASK mutations examined that were convincingly predicted by both sequence- (Table 1) and structure-based (Table 2) algorithms to disrupt CASK’s structure, results that were then easily confirmed in cell culture (Figure 3). These residues are close to one another in the folded protein (Figure 2D); W919, at the very C-terminal of CASK, rests in a β-strand that completes the fold of the split SH3 domain, in close proximity to Y728 (Figure 2D) [56]. These two radical mutations occur at highly conserved sites (Figure 1B) that are relatively buried and likely critical to stabilization of the protein fold. Our computational analyses suggest that the pathological effects of these mutations are likely due to destabilization of the protein structure (Tables 1 & 2).

Both GFP-CASK W919R and Y728C mutations formed protein aggregates that cluster near the nucleus (Figure 3). The aggregates are cytosolic in nature, as demonstrated by their co-localization with cytosolic mCherry (Figure 4). Computational algorithms identified two short regions of the CASK primary sequence that might contribute to amyloid fibril formation, but thioflavin T staining confirmed that the aggregates were not amyloid in nature (Figure 4). Neither did the aggregates exhibit substantial ubiquitination with immunostaining or under in vitro conditions since higher molecular weight bands were not observed for GFP-CASK-Y728C or W919R in Western blots (data not shown). It is possible that the aggregates occur only in the conditions of cell culture, where the overexpression of protein is, in this case, driven by the strong CMV promoter; in an organism possessing a CASK mutation that causes structural instability, such as Y728C or W919R, the protein would likely be cleared by normal cellular mechanisms before large amounts of unstable protein are produced and can accumulate to produce aggregates. The presence of the GFP fusion protein at the N-terminus of CASK allows detection of any unstable variants since GFP will fold effectively and remain stable [82].

Because CASK mutations are relatively rare, it is not yet possible to draw definitive conclusions about links between distinct clinical findings and specific mutations. Clinical phenotypes suggest that the domain in which the mutation is located may influence the severity of intellectual disability and the presence or absence of nystagmus, for example [8], [83]. Similarly, there are not enough data to suggest that pathogenesis associated with CASK mutations classified as “functional” differs substantially from that observed in individuals with mutations that likely disrupt structure. Generalized loss of CASK function, whether through disruption of protein-protein interactions or through absence of protein, is likely responsible for the developmental and intellectual disabilities seen in all individuals with CASK mutations. An important distinction between functional and misfolding CASK mutants lies in the possible therapeutic approaches to be pursued. As shown in this study, misfolding mutants could potentially be stabilized by employing folding chaperones. Here we have demonstrated that the protein aggregation observed with CASK-Y728C and CASK-W919R can be greatly reduced by the addition of glycerol (Figure 6). Glycerol has been used as a small molecule chemical chaperone to assist in the folding of proteins with misfolding mutations such as the cystic fibrosis protein, CFTR [65], among others (reviewed in [67]). Glycerol is an effective and widely used chaperone for in vitro studies because it both stabilizes proteins in their folded state by increasing the relative hydration of a protein [84] and prevents protein aggregation by stabilizing aggregation-prone folding intermediates [85]. It has also been demonstrated to induce innate molecular chaperones in vivo [86]. In cells expressing Y728C-GFP-CASK or W919R-GFP-CASK, glycerol treatment dramatically reduces the presence of protein aggregates. The effect of glycerol on these CASK mutants offers the tantalizing possibility that, for at least those XLMR CASK mutations that cause folding defects, there are therapeutic options that could be explored [87]. Based on cell viability studies (Figure S6), it is obvious that glycerol itself is not a suitable therapeutic option, and we are not suggesting this as an approach. The elimination of aggregates after glycerol addition, however, provides proof of concept and supports the pursuit of small molecules that are well-tolerated in vivo and stabilize CASK protein containing a misfolding mutation, as has been proposed for other proteins with known missense mutations such as the breast cancer gene BRCA1 [88]. This imaging-based cell assay could be used to quickly screen a wide variety of chaperone candidates for effectiveness at preventing aggregation that warrant further characterization in cell culture and animal models. Whether these CASK mutants, once stabilized, would function normally, remains to be evaluated.

The expression of the CASK variants in cell culture allowed us to evaluate the benefits of using numerous computational approaches to predict the impact of point mutations in this particular protein. Because each mutation studied has already been shown to associate with the XLMR pathology, it was hoped that the computational approaches would provide insight both into the possible nature of each mutation and into how best to interpret future computational results from sequence variants that have been identified but have not yet been directly associated with any pathology. Unfortunately, there was little consistency in the results generated by the computational approaches (see Table 1 and 2). On the other hand, a simple examination of sequence conservation (Figure 1B) turned out to be highly informative and predictive; the most conserved residues examined, Y728C and W919R, caused the most dramatic cellular phenotypes (Figure 3). Computational techniques that relied on either just sequence (Table 1) or sequence and structure (Table 2) more consistently predicted a pathological consequence for these two mutations than at the remaining three sites. There were, however, still computational methods that failed to identify Y728C and W919R as “pathological” or “destabilizing,” despite experimental evidence to the contrary. For the remaining mutations (R28L, Y268H, and P396S), the same simple examination of sequence conservation (Figure 1B) was similarly informative; from the observation that some CASK orthologs actually contain the mutant residue, it is natural to infer that the R28L mutation and the P396S mutation are unlikely to cause misfolding. The variability at position 268 suggests that mutation at this site is unlikely to disrupt protein fold since a range of residues is tolerated. For these three mutations, the computational methods, when taken in aggregate, were inconclusive; there was no consistent pattern that would allow one to definitively conclude that any of these mutations are, in fact, disease-causing. This suggests that exclusive reliance on a single or even multiple computational methods to predict the impact of a recently discovered sequence variant could lead to the wrong conclusion. There is still a convincing need for experimental verification to determine whether a putative mutation will lead to aberrations in protein structure or function and thus a disease state.

Each of the bioinformatics methods employed here has been thoroughly characterized and validated in other studies, [14]. The results presented here are not indicative of the overall performance of any particular method. Our results simply suggest that it is important to avoid drawing conclusions about a novel sequence variation from the results of a single approach. Potapov et al [89] did a comprehensive study of computational methods that predict ΔΔG changes in a protein upon point mutation and concluded that even when structural information is available, accurately predicting the change in ΔG for an individual protein remains challenging with existing approaches; trends were typically accurate, but any prediction of a single mutation should be interpreted with caution.

In general, bioinformatics approaches were useful at identifying the mutations that had the most dramatic impact on overall protein structure, such as Y728C and W919R. Preliminary identification of these mutations as damaging, however, was possible without any analysis beyond a simple alignment of orthologs. The combination of computational and cellular-based analyses allowed us to classify five known XLMR mutations as either structural or functional defects. For the three CASK mutations that showed no obvious cellular phenotype and were not clearly categorized as pathogenic with bioinformatics, the question remains as to the nature of their deleterious impact. The Y268H and P396S CASK mutations did segregate with affected individuals [8] but were identified only in individual families, leaving open the possibility of a chance disease association with a benign sequence variant. Future studies will address other possible functional defects such as altered protein-protein interactions with binding partners other than liprin-α or Mint-1 or signaling deficits. Each of these residues is highly conserved in chordates (Figure 1B) but can vary outside the phylum to incorporate the amino acid that causes human pathogenesis, suggesting a critical functional role that helps distinguish chordates from other organisms. The HEK-293 expression system provides a simple system for functional analyses of these and more recently identified mutations such as G197R [13].

Although identifying mutations responsible for a particular disorder is informative, it is merely the first step. Only after the nature of each mutation is elucidated can potential therapeutic approaches be explored. Our combination of simple bioinformatics analysis and cellular studies contributes to an understanding of why five specific mutations in CASK result in a range of developmental disorders characterized by structural defects in the brain. Our results form the basis for the development of a high-throughput screen to quickly identify misfolding mutants and screen a variety of small molecules for chaperoning capability. There are relatively few methodologies to detect protein stability in situ despite the clear need [90], [91]. In our approach, a protein of interest fused to a fluorescent protein at the N-terminal is expressed in cells driven by a strong promoter. Fusion at the N-terminal ensures folding of the fluorescent protein marker even if the protein of interest is unstable. A strong promoter results in accumulation of the unstable protein at a rate that likely cannot be accommodated by the cell’s proteasome machinery, allowing easy observation of the accumulated protein either with a fluorescent microscope or plate reader. This provides a simple, sensitive, and rapid way to identify misfolding mutants. It also serves as a platform to screen for small molecules that could be developed as therapeutic chaperones.

A personalized medicine approach is being taken for the treatment of diseases such as cystic fibrosis [92] and cancer [93], and in the future, therapeutic strategies to minimize the impact of hCASK mutations on a developing individual might be tailored to the implicated mutation type, either structural or functional. Promising results shown here indicate that for two of the CASK mutations (Y728C and W919R), addition of a chaperone agent such as glycerol might restore folded protein; future studies will be needed to determine whether CASK with these point mutations is functional once properly folded. In the case of a specific cystic fibrosis-associated mutation, simply increasing the amount of successfully folded mutant CFTR improves chloride conductance in cultured cells, suggesting that the particular misfolding mutation does not abolish protein function [65] but rather amount of available protein. In the case of CASK, it is intriguing to speculate about the possibilities of a similar approach for misfolding mutations. Perhaps the neurodevelopmental impact of CASK mutations could be minimized or even eliminated if enough properly folded CASK could be restored early in an individual’s life.

## Supporting Information

Figure S1 **List of sequences used in CASK multiple sequence alignments.** 30 sequences were identified by the ConSurf algorithm based on the sequence for hCASK (NP_003679.2). These sequences were used for generating multiple sequence alignments with ConSurf and ClustalOmega.
(TIF)

Figure S2 **Site-directed CASK mutagenesis** *A.* Reaction mixture for performing mutagenesis with Phusion® Kit. *B.* Cycling conditions for the mutagenesis PCR reactions. *C.* Primer sequences used for mutagenesis.
(TIF)

Figure S3 **High resolution image of cell with wildtype GFP-CASK.** HEK cells transfected with GFP-CASK and mCherry were imaged at 72 hours post-transfection. GFP-CASK displays a diffuse localization throughout the cell, excluding the nucleus (Nu).
(TIF)

Figure S4 **Solubility of wildtype CASK and mutants Y728C and W919R.** Cells transfected with wildtype CASK or GFP-CASK-Y728C or W919R were collected, lysates prepared, and blots run as described in Methods, except that lysate buffer did not contain Triton-X. Both the supernatant (soluble) and cell pellet were blotted. CASK and its mutants were found in both the soluble and pelleted fraction.
(TIF)

Figure S5 **The effect of extended incubation on aggregation propensity.** Cells transfected with all five CASK mutants were imaged 72 hours after transfection, rather than 20 hours after transfection. Extended incubation resulted in increased aggregation with the Y728C and W919R forms of CASK, but the other three mutants (R28L, Y268H, and P396S) showed no propensity to aggregate.
(TIF)

Figure S6 **Cell viability decreased with glycerol.** HEK cells were grown in 24-well plates with and without 10% glycerol. After 20 hours, live and dead cells were counted individually using Trypan blue exclusion. In wells in which 10% glycerol had been added, there were 20% fewer live cells than in untreated wells (p = .053, triplicates).
(TIF)

Figure S7 **Glycerol treatment eliminates intracellular aggregates.** Six hours after transfection, media was exchanged for either fresh media alone or containing 10% glycerol. Images, 20X.
(TIF)
