# Supplementary figures and images for: Identification and Glycerol-Induced Correction of Misfolding Mutations in the X-Linked Mental Retardation Gene CASK
Source: PLoS One. 2014 Feb 5;9(2):e88276. doi: 10.1371/journal.pone.0088276 (PMC3914952; doi:10.1371/journal.pone.0088276)

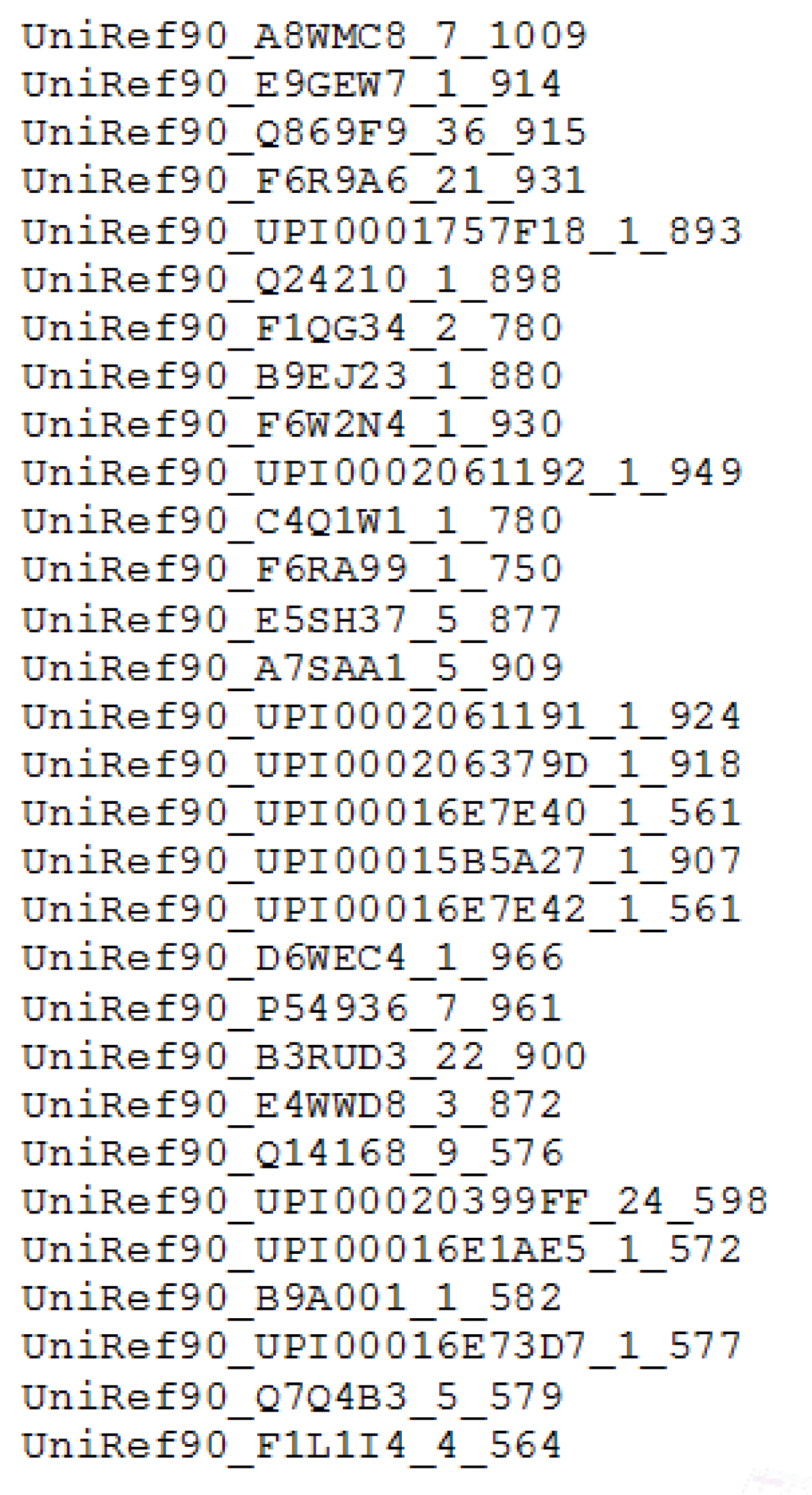

Supplement: Figure S1 — List of sequences used in CASK multiple sequence alignments. 30 sequences were identified by the ConSurf algorithm based on the sequence for hCASK (NP_003679.2). These sequences were used for generating multiple sequence alignments with ConSurf and ClustalOmega. (TIF) [file pone.0088276.s001.tif]

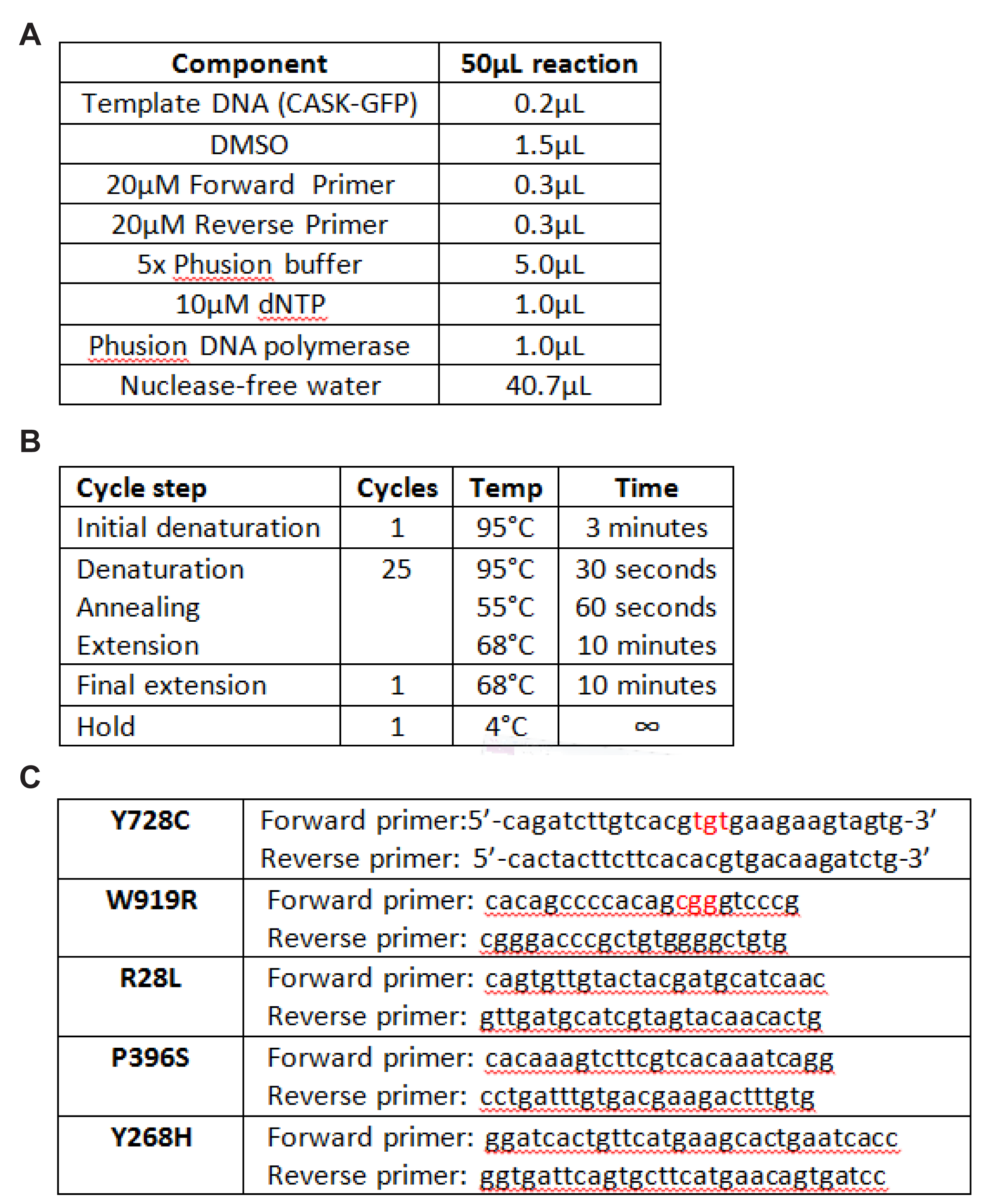

Supplement: Figure S2 — Site-directed CASK mutagenesis A. Reaction mixture for performing mutagenesis with Phusion® Kit. B. Cycling conditions for the mutagenesis PCR reactions. C. Primer sequences used for mutagenesis. (TIF) [file pone.0088276.s002.tif]

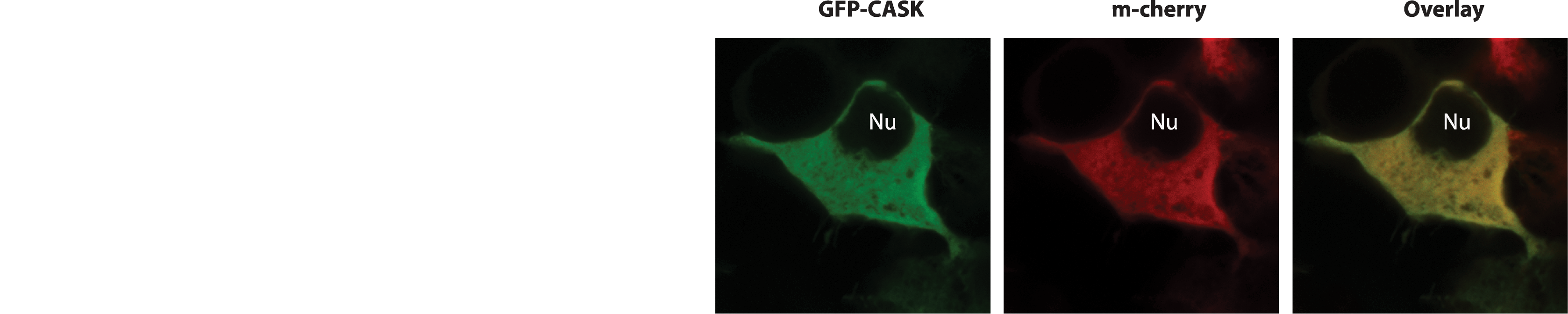

Supplement: Figure S3 — High resolution image of cell with wildtype GFP-CASK. HEK cells transfected with GFP-CASK and mCherry were imaged at 72 hours post-transfection. GFP-CASK displays a diffuse localization throughout the cell, excluding the nucleus (Nu). (TIF) [file pone.0088276.s003.tif]

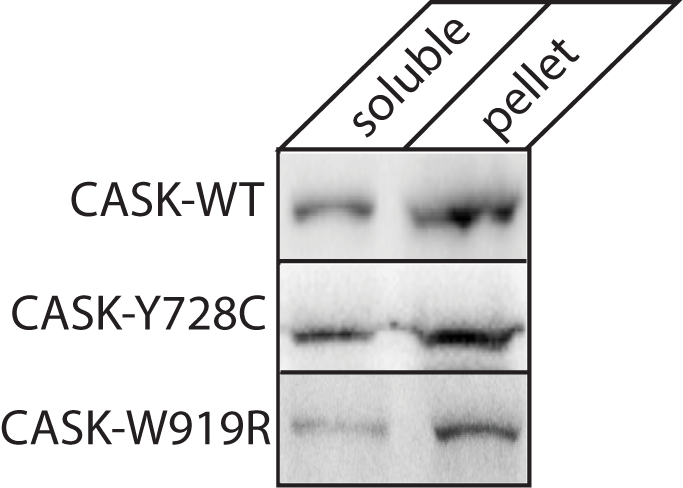

Supplement: Figure S4 — Solubility of wildtype CASK and mutants Y728C and W919R. Cells transfected with wildtype CASK or GFP-CASK-Y728C or W919R were collected, lysates prepared, and blots run as described in Methods, except that lysate buffer did not contain Triton-X. Both the supernatant (soluble) and cell pellet were blotted. CASK and its mutants were found in both the soluble and pelleted fraction. (TIF) [file pone.0088276.s004.tif]

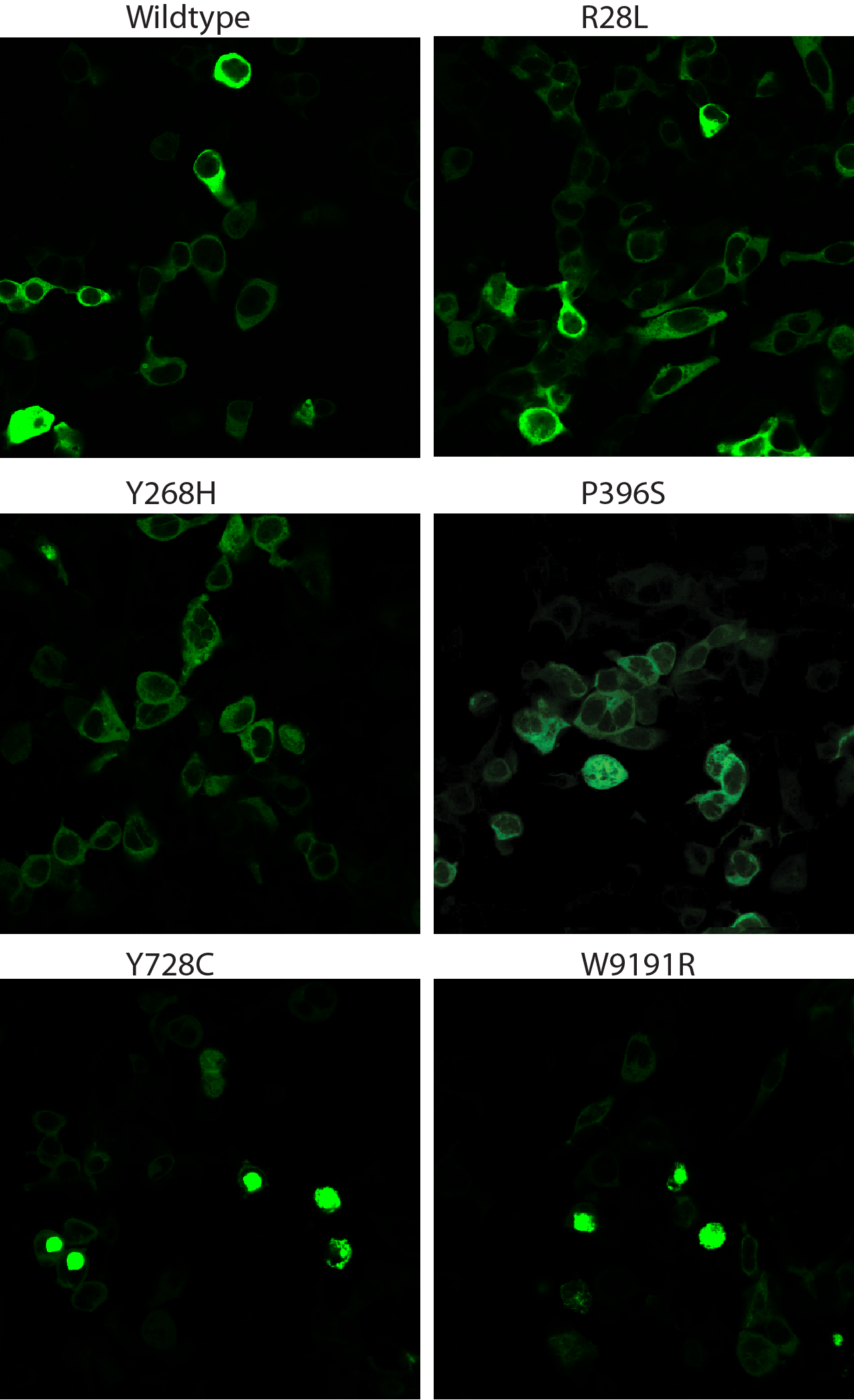

Supplement: Figure S5 — The effect of extended incubation on aggregation propensity. Cells transfected with all five CASK mutants were imaged 72 hours after transfection, rather than 20 hours after transfection. Extended incubation resulted in increased aggregation with the Y728C and W919R forms of CASK, but the other three mutants (R28L, Y268H, and P396S) showed no propensity to aggregate. (TIF) [file pone.0088276.s005.tif]

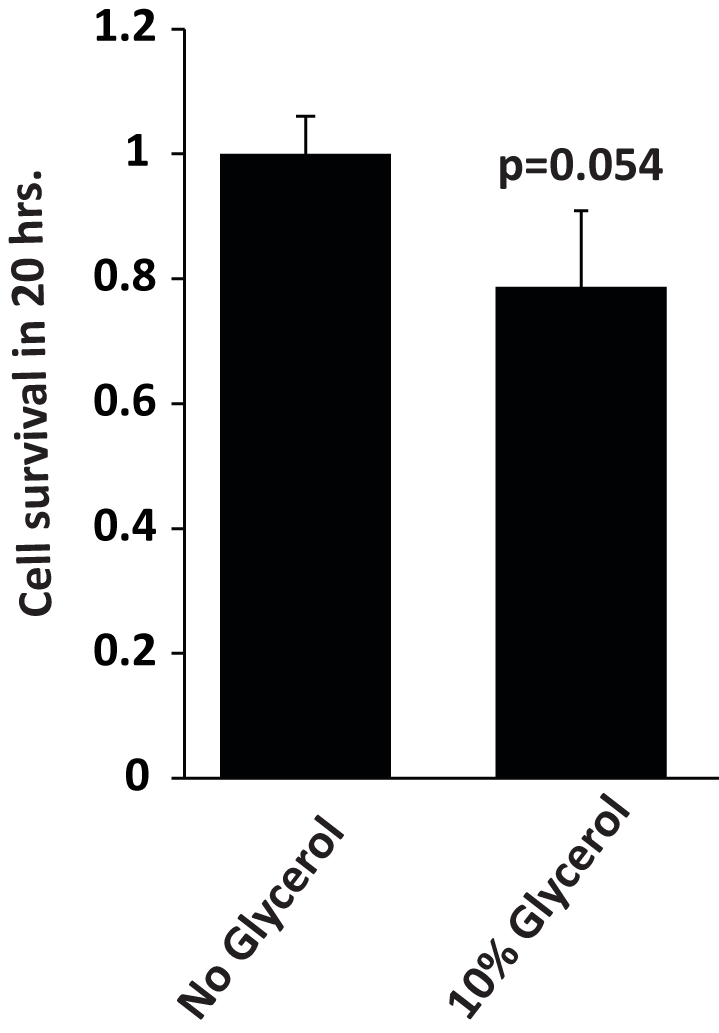

Supplement: Figure S6 — Cell viability decreased with glycerol. HEK cells were grown in 24-well plates with and without 10% glycerol. After 20 hours, live and dead cells were counted individually using Trypan blue exclusion. In wells in which 10% glycerol had been added, there were 20% fewer live cells than in untreated wells (p = .053, triplicates). (TIF) [file pone.0088276.s006.tif]

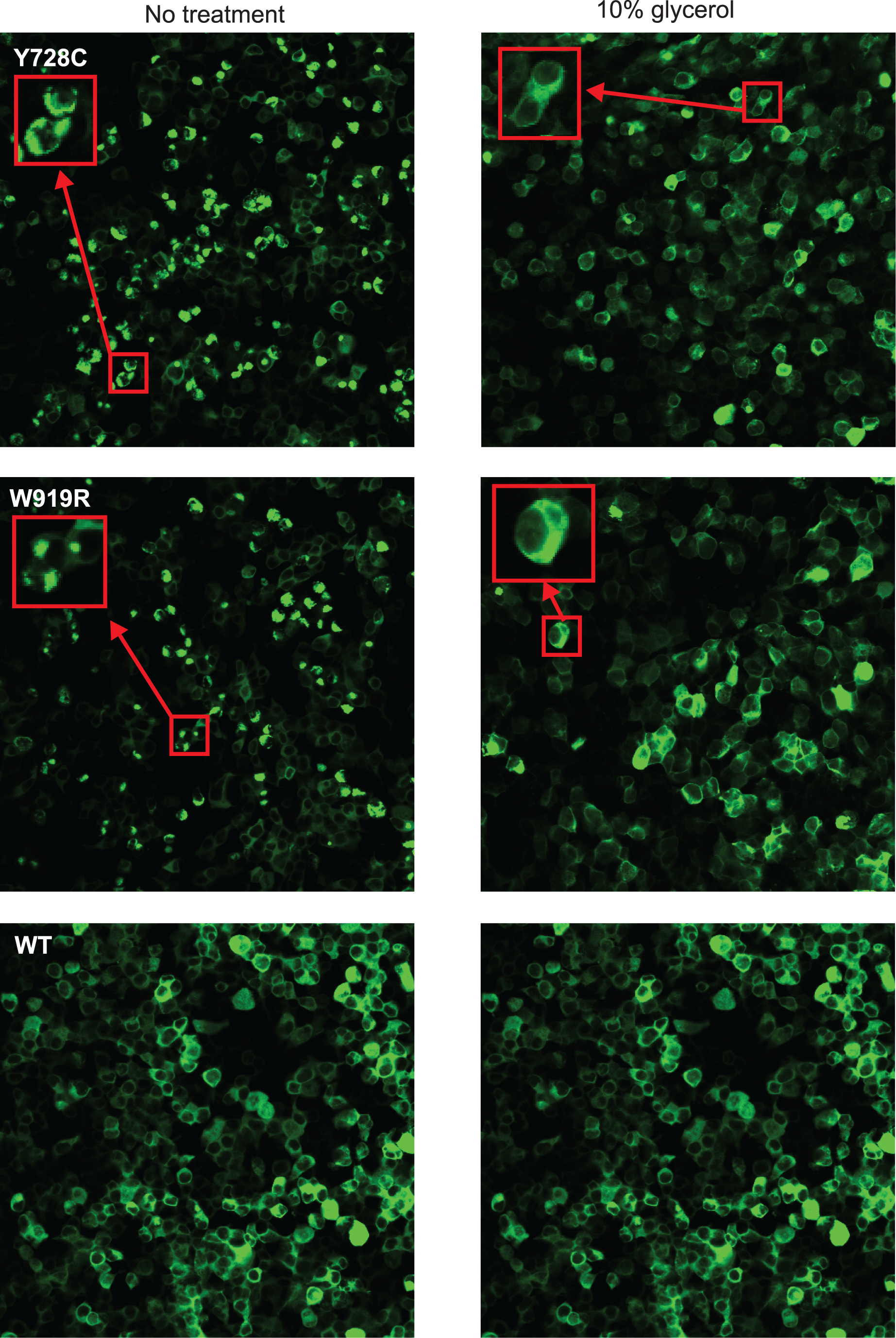

Supplement: Figure S7 — Glycerol treatment eliminates intracellular aggregates. Six hours after transfection, media was exchanged for either fresh media alone or containing 10% glycerol. Images, 20X. (TIF) [file pone.0088276.s007.tif]
